# Tomosyn Negatively Regulates Arginine Vasopressin Secretion in Embryonic Stem Cell-Derived Neurons

**DOI:** 10.1371/journal.pone.0164544

**Published:** 2016-10-12

**Authors:** Seiji Takeuchi, Shintaro Iwama, Hiroshi Takagi, Atsushi Kiyota, Kohtaro Nakashima, Hisakazu Izumida, Haruki Fujisawa, Naoko Iwata, Hidetaka Suga, Takashi Watanabe, Kozo Kaibuchi, Yutaka Oiso, Hiroshi Arima, Yoshihisa Sugimura

**Affiliations:** 1 Department of Endocrinology and Diabetes, Nagoya University Graduate School of Medicine, Nagoya, Japan; 2 Department of Cell Pharmacology, Nagoya University Graduate School of Medicine, Nagoya, Japan; Augusta University, UNITED STATES

## Abstract

Arginine vasopressin (AVP) is secreted via exocytosis; however, the precise molecular mechanism underlying the exocytosis of AVP remains to be elucidated. To better understand the mechanisms of AVP secretion, in our study we have identified proteins that bind with a 25 kDa synaptosomal-associated protein (SNAP25). SNAP25 plays a crucial role in exocytosis, in the posterior pituitary. Embryonic stem (ES) cell-derived AVP neurons were established to investigate the functions of the identified proteins. Using glutathione S-transferase (GST)-pulldown assays and proteomic analyses, we identified tomosyn-1 (syntaxin-binding protein 5) as a SNAP25-binding protein in the posterior pituitary. Coimmunoprecipitation assays indicated that tomosyn formed N-ethylmaleimide-sensitive factor attachment protein receptor (SNARE) complexes with SNAP25 and syntaxin1. Immunohistochemistry showed that tomosyn localized to the posterior pituitary. Mouse ES cells self-differentiated into AVP neurons (mES-AVP) that expressed tomosyn and two transmembrane SNARE proteins, including SNAP25 and syntaxin1. KCl increased AVP secretion in mES-AVP, and overexpression of tomosyn-1 reduced KCl-stimulated AVP secretion. Downregulation of tomosyn-1 with siRNA increased KCl-stimulated AVP secretion. These results suggested that tomosyn-1 negatively regulated AVP secretion in mES-AVP and further suggest the possibility of using mES-AVP culture systems to evaluate the role of synaptic proteins from AVP neurons.

## Introduction

Arginine vasopressin (AVP) is a hormone involved in maintaining fluid homeostasis. AVP is synthesized primarily in the magnocellular neurons of the supraoptic nucleus (SON) and paraventricular nucleus (PVN) in the hypothalamus [1]. After axonal transport, AVP vesicles are stored in the nerve terminal of the posterior pituitary, and AVP is secreted by exocytosis when an action potential depolarizes the nerve terminal [1].

The process of exocytosis of AVP-containing vesicles, as well as of neurotransmitters and hormones, is thought to involve several steps, including recruitment, docking, priming, and fusion [2, 3]. Soluble N-ethylmaleimide-sensitive factor attachment protein receptor (SNARE) proteins play a crucial role in exocytosis of neurotransmitters and hormones such as insulin [4–7]. During membrane fusion, one vesicular SNARE protein, synaptobrevin/vesicle associated membrane protein (VAMP), and two transmembrane SNARE proteins including soluble N-ethylmaleimide attachment protein-25 (SNAP25) and syntaxin1, combine to form a trans-SNARE complex [8]. These SNARE proteins are expressed in the hypothalamo-neurohypophysial system (HNS) [9]. In addition, other SNARE proteins, including synaptotagmin, a Ca^2+^-dependent activator protein for secretion (CAPS-1), mammalian uncoordinated-18 (Munc-18), a cysteine string protein (CSP), α-SNAP [soluble N-ethylmaleimide-sensitive factor (NSF) attachment protein], and ras-related protein (Rab-3A), are expressed in the HNS [9–15]. Recently, we have reported that rabphilin-3A, a rab3a effector protein, might be a major autoantigen in infundibulo-neurohypophysitis (LINH), which causes central diabetes insipidus (CDI) due to insufficient AVP secretion, and autoantibodies against rabphilin-3A might be diagnostic markers for LINH [16]. However, the precise molecular mechanism underlying the exocytosis of AVP remains to be elucidated. One reason may be that cell culture systems used to investigate the molecular mechanisms of AVP secretion by knockdown or overexpression experiments have not been established. Many reports on AVP secretion from a variety of preparations (isolated hypothalamo-neurohypophysial systems, isolated posterior pituitaries, and isolated nerve endings) have shown the involvement of extracellular molecules such as ATP, osmotic agents (sodium and mannitol), high potassium, GABA, and other neuropeptides in AVP secretion or electrophysiological effects in nerve terminals of the posterior pituitary [17–22]. Jurgutis et al. reported that SNARE proteins, including SNAP25, syntaxin, and synaptobrevin were present in isolated nerve ending of rat neurohypophysis and that synaptobrevin 2 played an important role in exocytotic release, using membrane capacitance measurements together with Botulinum B toxin [23]. However, to the best of our knowledge, there have been no reports that provide direct evidence for the involvement of cellular molecules (for example, the proteins that are known to be involved in the trafficking or exocytosis, including SNARE proteins) in the release of hormones, including vasopressin and oxytocin from the neurohypophysis using knockdown or overexpression in previous culture systems. It has been reported, by using synaptotagmin IV knockout mice, that synaptotagmin IV is a multifunctional regulator of peptidergic nerve terminals [24]. However, alterations in the release of the neuropeptides were not shown. Embryonic stem (ES) cells have great potential not only for regenerative medicine studies but also for biological studies. Recently, it has been reported that ES cells differentiate in serum-free medium (SFEBq media) into rostral hypothalamic progenitor cells, including AVP-secreting neurons, with embryoid body-like aggregates that quickly reaggregate in growth factor-free chemically defined medium (gfCDM) [25].

In the present study, using proteomics analyses, we identified proteins that bind with SNAP25 in the posterior pituitary. We also developed mouse embryonic stem (ES) cell-derived AVP neurons to investigate the functions of the identified synaptic protein.

## Results

### Tomosyn binds to SNAP25 in the posterior pituitary

**Fig 1:**
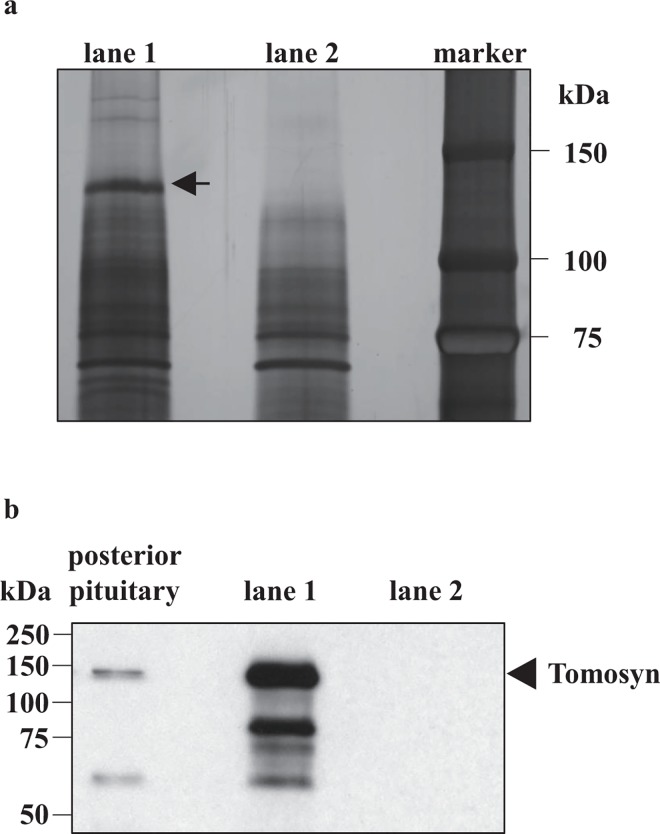
Tomosyn is a SNAP25 binding protein in the posterior pituitary. (a) *In vitro* binding assay. Rat posterior pituitary lysates was incubated with GST-SNAP25 and glutathione (GSH) Sepharose. Eluted proteins bound to the beads were separated on SDS-PAGE followed by silver staining of proteins. The arrow indicates the band of interest. (b) Western blotting of pull-down samples. The arrowhead indicates the location of the immune reactive band reacting with anti-tomosyn antibody (Santa Cruz Biotechnology #sc-136105). Lane 1 = GST-SNAP25 with rat posterior pituitary lysates; lane 2 = GST-SNAP25 without rat posterior pituitary lysates (for both a, and b); Marker = molecular weight markers.

To search for binding proteins to SNAP25, glutathione S-transferase (GST) pulldown assays were performed using GST-SNAP25 and rat posterior pituitary lysates. The eluted proteins were subjected to sodium dodecyl sulphate polyacrylamide gel electrophoresis (SDS-PAGE) followed by silver staining. Consequently, a band migrating at a molecular weight of approximately 130 kDa was detected in the eluted sample (Fig 1A). The band migrating at a molecular weight of approximately 130 kDa was not detected in the eluted samples using GST-cdc42-GTPγS beads with rat posterior pituitary lysates (S1 Fig), strongly suggesting that the possibility of nonspecific binding of proteins in the lysates to the beads or GST tag was excluded. As this 130 kDa protein was thought to be a significant candidate, this band was then subjected to in-gel digestion and nano-liquid chromatography–mass spectrometry/mass spectrometry (LC/MS/MS) analysis. As a result, tomosyn-1 (also known as syntaxin binding protein 5) was identified. Two isoforms including b-tomosyn-1 and m-tomosyn-1 were detected. The sequence coverages of b- and m-tomosyn-1 were 54.6% and 56.3%, respectively. Tomosyn-2 was not detected. We compared the mRNA expressions of b- and m-tomosyn-1 in rat hypothalamus and cortex and confirmed that the expression of m-tomosyn-1 was much higher than that of b-tomosyn-1 in the hypothalamus and cortex (data not shown). To verify the expression of tomosyn, the eluted samples were subjected to SDS-PAGE followed by western blotting using anti-tomosyn antibody. The presence of tomosyn in the pull down sample was confirmed (Fig 1B). This result verified the results of the proteomic analyses.

### Tomosyn localizes in the posterior pituitary

**Fig 2:**
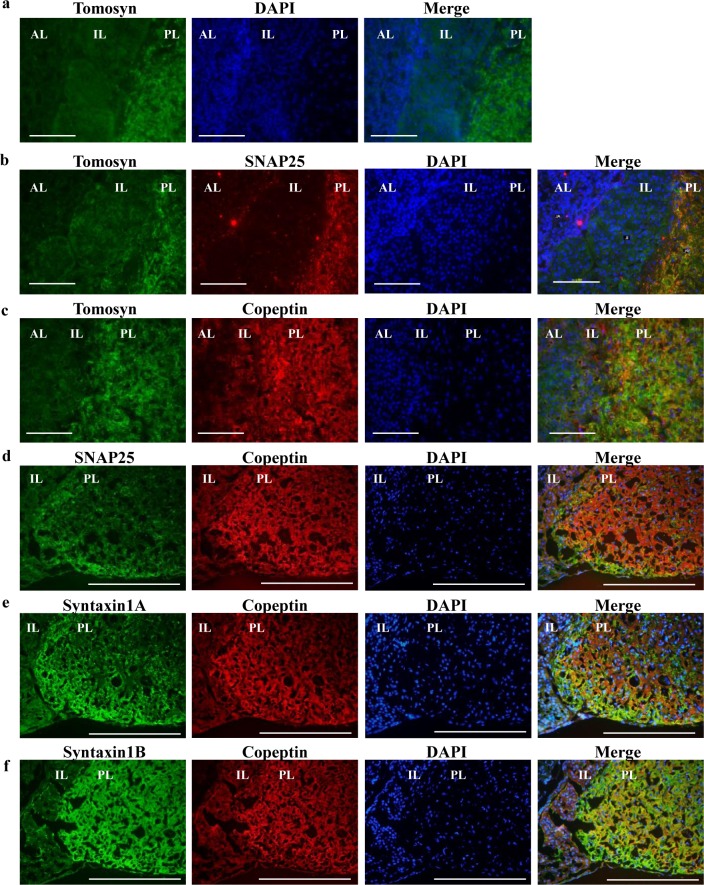
Tomosyn localizes in posterior pituitary. (a) The cryosections of rat posterior pituitary were single stained with anti-tomosyn or DAPI (labelled on the top). The merged image is show at the right. (b–f) Double stained images are shown and the antibodies used are shown on top each image. Merged images are shown at right. Scale bars indicate 100 μm (white bars). AL, anterior lobe of the pituitary; IL, intermediate lobe; and PL, posterior lobe.

Immunolocalization analyses showed that immunoreactivity for tomosyn, SNAP25, syntaxin1A, and syntaxin1B were all detected in the posterior pituitary (Fig 2A–2F). Immunoreactivity for tomosyn, SNAP25, syntaxin1A, and syntaxin1B was overlapped with that of copeptin, a marker of vasopressin (Fig 2C–2F). In addition, immunoreactivity for tomosyn was overlapped with that of SNAP25 (Fig 2B) that is known to be expressed in the posterior pituitary axon terminal [9, 23]. Because the cell number in the posterior pituitary is less compared with that in the anterior and intermediate pituitary, 4',6-diamidino-2-phenylindole (DAPI) staining of nuclei was weak in the posterior pituitary. In contrast, immunofluorescent labeling of tomosyn or SNAP25 was not detected in the supraoptic nucleus (SON) or the paraventricular nucleus (PVN) of the hypothalamus, while positive staining was observed for copeptin (positive control) (S2 Fig).

### Tomosyn is expressed in the rat hypothalamo-posterior pituitary axis at both protein and mRNA levels

**Fig 3:**
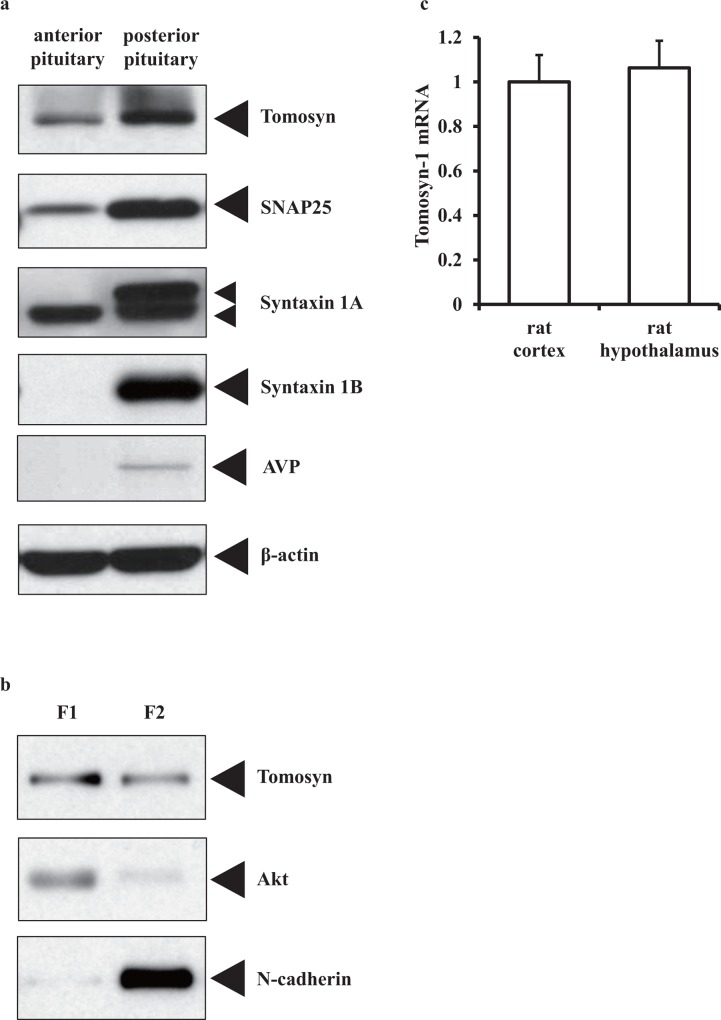
Tomosyn is expressed in the rat hypothalamo-posterior pituitary axis. (a) Presence of tomosyn in the rat anterior and posterior pituitary as indicated by western blotting. Pituitary lysates were separated on SDS-PAGE followed by western blotting using anti-tomosyn, anti-SNAP25, anti-syntaxin 1A, anti-syntaxin 1B, anti-AVP, and anti-β-actin antibodies (as a loading control). (b) Subcellular fractionation of rat posterior pituitary samples. Fraction 1 (cytosolic proteins, F1) and Fraction 2 (membranes and membrane organelles, F2) were subjected to SDS-PAGE followed by western blotting using anti-tomosyn antibody. Five μg of proteins was loaded onto each lane. The subcellular fractionation was confirmed by western blotting with anti-Akt (cytosolic marker) and anti-N-cadherin (membrane fraction marker) antibodies. Tomosyn was present in both fractions. (c) Expression levels of tomosyn-1 mRNA in rat cortex and hypothalamus are similar. The amount of mRNA was determined using quantitative RT-PCR. The values are normalized to β-actin mRNA and are expressed as the mean ± SEM.

Next, tomosyn protein expression was assessed by western blot analysis. Immunoblotting studies showed that tomosyn, SNAP25, syntaxin1A, and syntaxin1B were all expressed in the posterior pituitary as shown by western blotting analyses (Fig 3A). In addition, subcellular fractionation demonstrated that tomosyn localized in both cytosolic (F1) and membrane fractions (F2) (Fig 3B). The mRNA expression was evaluated by reverse transcription polymerase chain reaction (RT-PCR) analyses. Tomosyn-1 mRNA expression in rat hypothalamus was confirmed at the same level as was observed in rat cortex (Fig 3C).

### Tomosyn forms SNARE complexes with SNAP25 and syntaxin1 in the posterior pituitary

**Fig 4:**
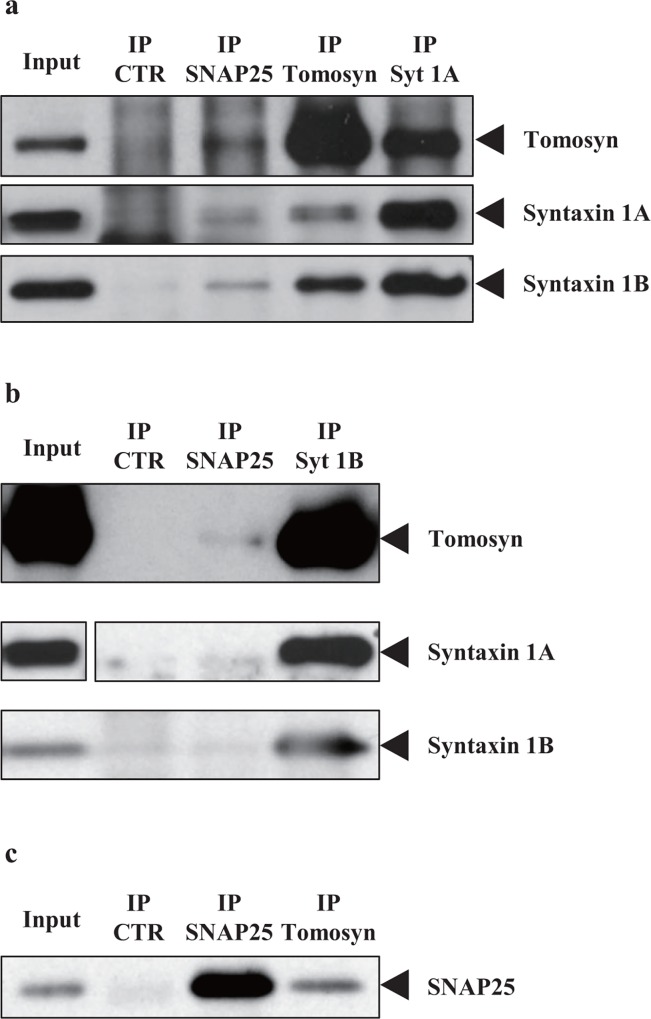
Tomosyn forms SNARE complexes with SNAP25 and syntaxin 1 in the posterior pituitary. (a–c) Coimmunoprecipitation assays were performed using protein G Sepharose and mouse immunoprecipitation (IP) antibodies (a), protein A Sepharose and rabbit IP antibody (b) or ImmunocruzE^™^ imaging system (Santa Cruz Biotechnology, Santa Cruz, CA, USA) and mouse IP antibody (c), respectively. Eluted samples were subjected to SDS-PAGE followed by western blotting using anti-tomosyn, anti-SNAP25, anti-syntaxin 1A, or anti-syntaxin 1B antibodies (arrowheads at right). Input was 1/200 (volume) of the total rat posterior pituitary lysates used for the assay. CTR = IP with anti-IgG antibody.

To investigate the interactions of tomosyn with SNAP25 and syntaxin-1 in the posterior pituitary, we performed coimmunoprecipitation assays using posterior pituitary lysates. As predicted, tomosyn coimmunoprecipitated with SNAP25, syntaxin 1A, (Fig 4A), and syntaxin1B (Fig 4B). In addition, SNAP25 (Fig 4C), syntaxin 1A, and syntaxin 1B (Fig 4A) also coimmunoprecipitated with tomosyn using their specific antibodies. Syntaxin 1A and syntaxin 1B were coimmunoprecipitated with each other, suggesting that syntaxin1A and 1B is in a hetero-oligomer. These data show that tomosyn forms SNARE complexes with SNAP25 and syntaxin1 in the posterior pituitary.

### AVP neurons from mouse ES cells express tomosyn

**Fig 5:**
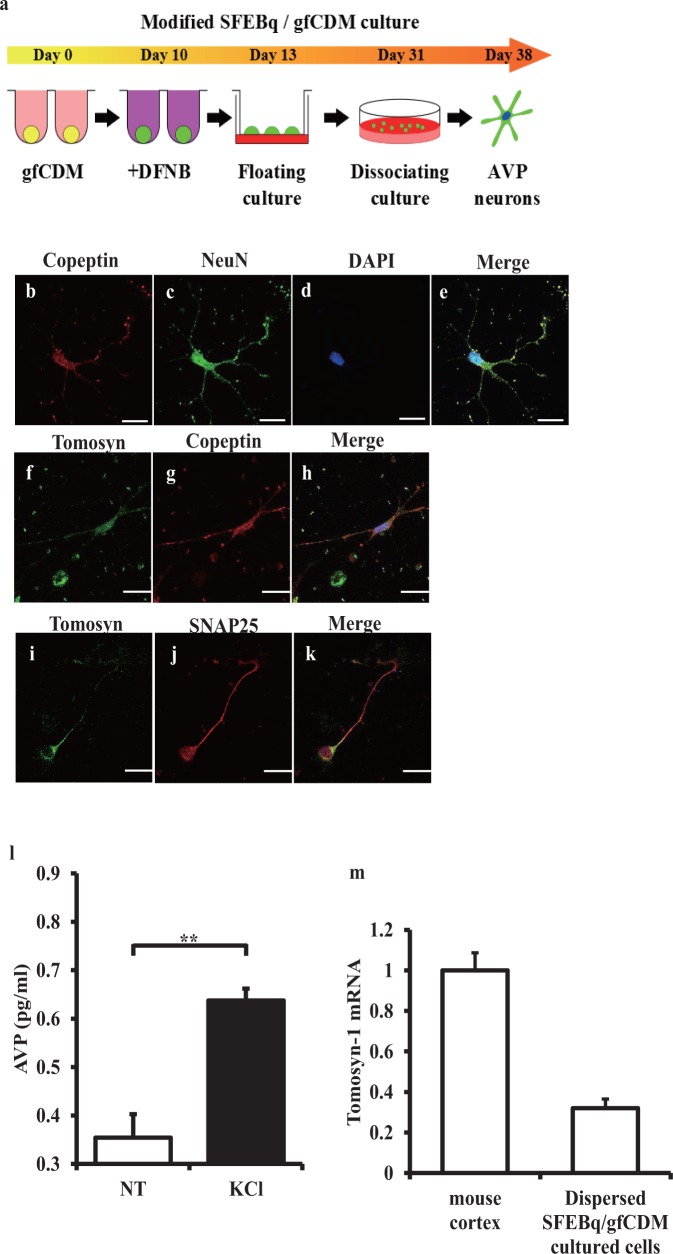
Arginine vasopressin (AVP)-secreting neurons from mouse embryonic stem cells express tomosyn. (a) Flow chart showing the method for culturing modified embryonic stem (ES) cells differentiating in serum-free medium (SFEBq)/growth factor-free chemically defined medium (gfCDM). DFNB = DMEM/F12 supplemented with 7 g/L glucose, N2 and B27. (b–e) Immunostaining with copeptin (red), NeuN (green), and DAPI (blue) in dispersed SFEBq/gfCDM cultured cells. A merged image is shown in the right panel. White scale bars indicate 20 μm. (f–k) Immunolocalization of proteins in dispersed SFEBq/gfCDM cultured cells, immunostaining with anti-tomosyn (green), anti-copeptin (red), or anti-SNAP25 (red) antibodies as analysed with confocal microscopy. Merged images are shown in the right panels. White scale bars indicate 25 μm. (l) AVP levels with or without 100 mM KCl stimulation in SFEBq/gfCDM cultured cells (see Methods section). AVP concentrations in the media of artificial cerebrospinal fluid cultured cells (aCSF) (non-treated, NT; n = 8), or 100 mM KCl treatment (KCl; n = 8) are shown. Values are expressed as the mean ± SEM. **P < 0.01 versus NT (non-treated artificial spinal fluid). (m) Tomosyn-1 mRNA expressions in dispersed SFEBq/gfCDM cultured cells. The amount of mRNA was determined using quantitative RT-PCR. The values are normalized to β-actin mRNA and are expressed as the mean ± SEM.

Wataya et al. reported differentiation from mouse ES cells into magnocellular vasopressinergic neurons [25]. With minor modifications, we constructed a dispersed culture system from SFEBq/gfCDM cultured aggregates, named mES-AVP cell cultures. The mouse ES cells (EB5) were cultured as floating aggregates in serum-free conditions for up to day 31 without cell sorting. SFEBq/gfCDM cultured aggregates were subsequently dissociated using an enzymatic reagent for isolation of nerve cells, and were then plated on gelatin-coated dishes (Fig 5A). The numbers of copeptin-positive and tomosyn-positive cells among the DAPI-positive cells were counted, and were 3.1% and 9.0%, respectively (data not shown). Immunofluorescent analyses showed copeptin-positive AVP cells also expressed NeuN, a neuronal marker, suggesting copeptin-positive AVP cells maintained their neuronal identity (Fig 5B–5E). Immunofluorescent analyses also showed that copeptin-positive AVP neurons expressed tomosyn (Fig 5F–5H) and that SNAP25 and tomosyn were costained in these neurons (Fig 5J and 5K). Fig 5L shows that AVP secretion was significantly stimulated by 100 mM KCl (P < 0.01) compared to artificial cerebrospinal fluid (NT) alone. In addition, tomosyn-1 mRNA expression was measured by RT-PCR (Fig 5M). These results demonstrated that ES cell-derived AVP neurons were successfully cultured.

### Tomosyn-1 negatively regulates AVP secretion

**Fig 6:**
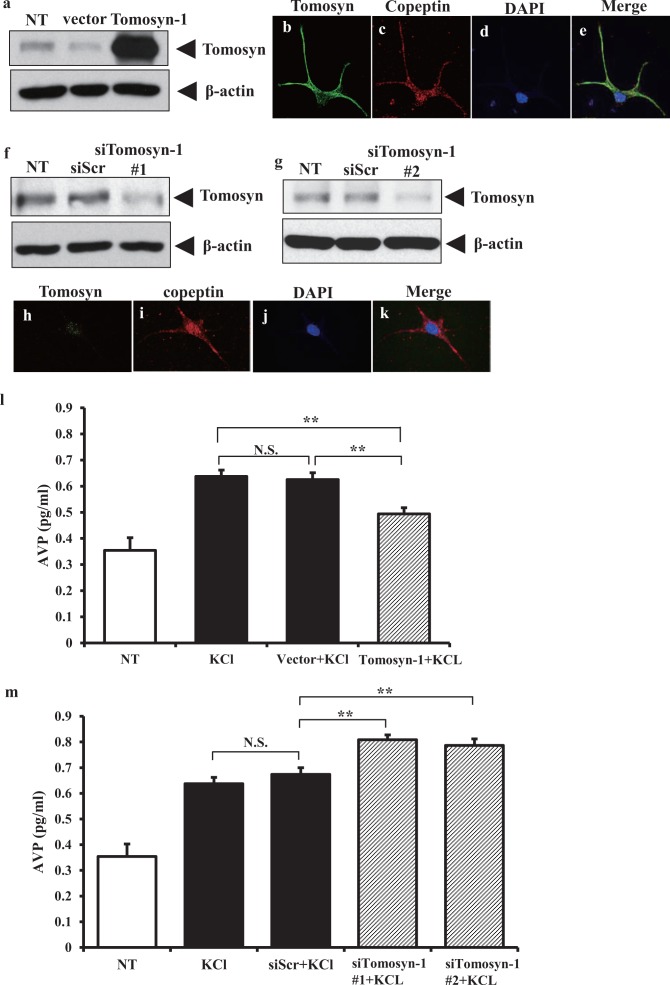
Tomosyn-1 negatively regulates secretion of AVP vesicles. (a–k) The effects of overexpression or siRNA treatments on tomosyn-1 expression. Dispersed SFEBq/gfCDM cultured cells were transfected with empty vector (vector) or tomosyn-1 vector (Tomosyn-1), scrambled siRNA (siScr) or Tomosyn-1 siRNA (siTomosyn-1 #1 or #2), or not treated (NT). (a, f, g) After 48 h, the expression levels of tomosyn were analysed by western blotting using anti-tomosyn antibody (Tomosyn, marked with arrowheads at the right). The levels of β-actin in the same samples were determined as a control for protein loading (bottom panels of a, f, g). (b–e) Representative immunostaining for tomosyn (green), copeptin (red), and DAPI (blue) at 48 h after overexpression of tomosyn-1 in dispersed SFEBq/gfCDM cultured cells. The merged image is shown in (e). (h–k) Representative immunostaining for tomosyn (green), copeptin (red), and DAPI (blue) at 48 h after knockdown of tomosyn-1 with siRNA in dispersed SFEBq/gfCDM cultured cells. The merged image is shown in (k). (l) AVP concentration in the media of artificial cerebrospinal fluid cultured cells (aCSF) (non-treated, NT; n = 13), KCl treatment (KCl; n = 13), empty vector with KCl (Vector + KCl; n = 12), and tomosyn-1 vector with KCl (Tomosyn-1 + KCl; n = 12). (m) AVP concentration from scrambled siRNA with KCl (siScr + KCl; n = 13), and siTomosyn-1 with KCl (siTomosyn-1 #1 + KCl group; n = 13, siTomosyn-1 #2 + KCl group; n = 12). Final KCl concentration was 100 mM. Values are expressed as the mean ± SEM. **P < 0.01.

To investigate the role of tomosyn-1 in AVP secretion, we performed overexpression or knockdown experiments using plasmid constructs, and measured AVP levels in media in mES-AVP cultures. Overexpression of tomosyn-1 and knockdown of tomosyn-1 with siRNA were confirmed by immunoblotting (Fig 6A, 6F and 6G). The immunoreactivity of tomosyn in copeptin-positive AVP neurons was increased (Fig 6B–6E) or reduced (Fig 6H–6K) with expression of tomosyn-1 and siTomosyn-1, respectively. In addition, cells expressing tomosyn-1 (Fig 6B–6E) and siTomosyn-1 (Fig 6H–6K) did not show significant morphological changes of copeptin-positive AVP neurons, suggesting that cell viability was not affected by the transfection procedure. Overexpression of tomosyn-1 significantly reduced KCl-stimulated AVP secretion (P < 0.01) (Fig 6L). In contrast, partial knockdown of tomosyn-1 significantly increased KCl-stimulated AVP secretion (P < 0.01) (Fig 6M). However, overexpression and knockdown of tomosyn-1 did not significantly affect AVP secretion (S3 Fig) in the absence of KCl stimulation. These results demonstrate that tomosyn-1 negatively regulates the depolarization-evoked AVP secretion in mES-AVP cells.

## Discussion

In this study, we have developed a method to identify proteins interacting with SNAP25 in the posterior pituitary. Combination of GST-pulldown assays and proteomic analyses revealed that tomosyn-1 was first identified as a binding protein of SNAP25 in the posterior pituitary. We developed a mES-AVP cell system to investigate the function of tomosyn-1 in AVP secretion. We found that tomosyn-1 negatively regulates AVP secretion in mES-AVP cells.

Immunofluorescence analyses showed that immunoreactivities for tomosyn were present in the posterior pituitary, but not in the SON and PVN of the hypothalamus. Fujita et al. identified tomosyn-1 as a syntraxin-1 binding protein that forms a complex with syntaxin-1, SNAP-25, and synaptotagmin [26]. They reported that tomosyn-1 was localized both on the plasma membranes and in the cytosol of the presynapse and that the distribution of tomosyn-1 overlapped with syntaxin-1 in nerve terminals in the brain. SNAP25 is localized to the cytosolic face of the plasma membrane where the sites of secretion, such as the active zone of the synapse exist [27–29]. Tomosyn-1 binds with syntaxin-1 and SNAP25 [26, 30, 31]. Tobin et al. reported that SNAP25 is not present in the somata and dendrite of SON neurons and is colocalized with the vasopressin peptide in the terminal axons of the posterior pituitary [9]. In agreement with their report, in our study, immunoreactivities for tomosyn, SNAP25, syntaxin1A, and syntaxin1B were detected in the posterior pituitary. In addition, immunoreactivity for tomosyn was overlapped with that of SNAP25, and neither tomosyn nor SNAP25 was detected in the SON and PVN in the hypothalamus. Taken together, our results suggest that endogenous tomosyn interacts with endogenous SNAP25, and that tomosyn is also localized in the posterior pituitary where SNAP25 is localized. However, more data are required using direct *in situ* experiments (i.e. using electron microscopy) to measure the interactions of tomosyn and SNAP25.

Tomosyn-1 consists of two domains: a C-terminal VAMP-like domain (VLD) and a large N-terminal region that contains WD40 repeats [32, 33]. Although we did not examine the domain of tomosyn-1 responsible for binding with SNAP25, using coimmunoprecipitation assays we confirmed that endogenous tomosyn bound with endogenous syntaxin-1 and SNAP25 in rat posterior pituitary. The VLD of tomosyn-1 competes with VAMP-2 to form nonfusogenic tomosyn-1 SNARE complexes, thereby decreasing exocytosis in PC12 cells [32, 33]. In addition, N-terminal WD40 repeats of tomosyn-1 negatively regulate the secretion of neurotransmitters by enhancing the oligomerization of the SNARE complex and by inhibiting the function of the calcium sensor synaptotagmin-1 [34, 35].

Although we identified tomosyn-1 as a SNAP25-binding protein, Yu et al. recently reported that tomosyn-1 binds strongly to the syntaxin1-SNAP25 complex and blocks VAMP2 association [36]. We showed that tomosyn bound to syntaxin1 or SNAP25 individually by coimmunoprecipitation assays using posterior pituitary in the present study. In the future, we need to clarify whether tomosyn-1 binds to the syntaxin1-SNAP25 complex in AVP neurons, and the role of the N- and C- terminal domains of tomosyn-1 in SNARE complex formation.

To investigate the function of tomosyn-1 in AVP secretion, we designed an ES-AVP cell culture system. Previously, there have been no appropriate AVP cell lines to investigate AVP secretion. Wataya et al. reported, by using SFEBq/gfCDM cultures that ES cells differentiate into rostral hypothalamic progenitor cells, including AVP secreting neurons [25]. Modifying their original method of SFEBq/gfCDM culturing, we constructed a dispersed culture system from SFEBq/gfCDM cultured aggregates. The percentages of copeptin- and tomosyn-positive cells among the DAPI-positive cells were 3.1% and 9.0%, respectively. Regarding the percentage of copeptin-positive cells in ES AVP culture systems, Wataya et al. reported that AVP-positive neurons were found in aggregates and represented 6.0% of the total population of cells on day 20 [25]. However, they did not show the percentage of copeptin-positive cells in dispersed cultures from the aggregates. In the same study, FACS sorting (on day 7) of Rax (specifically expressed in the rostral hypothalamic neuro-epithelial-GFP progenitors from SFEBq/gfCDM-cultured ES cells) was done. Previously, we showed that mouse ES cells were cultured as floating aggregates in serum-free conditions for up to 31 days without cell sorting. The SFEBq/gfCDM cultured aggregates were subsequently dissociated using an enzyme. Therefore, the difference of the percentage of copeptin-positive cells in ES AVP culture systems might be partially due to the difference in methodology. Regarding the percentage of tomosyn-positive cells, it is possible that some types of neuron or neuroendocrine cells other than vasopressin neurons also express tomosyn in ES AVP culture systems. This might be one reason why the percentage of tomosyn positive cells was larger than that of copeptin-positive cells in ES AVP culture systems. In addition to determining the percentages of tomosyn-positive cells or mRNA expression of tomosyn-1 in ES AVP cultures, it will also be necessary to identify the types of neuroendocrine cells or neurons expressing tomosyn, and the percentage of those cells expressing tomosyn *in vivo* in the hypothalamus. The dispersed culture system had a neuron-like immunoreactivity profile, with AVP-positive cells that were immune positive for tomosyn and SNAP25. The immunoreactivity of tomosyn was detected in the axon-like process, which is consistent with the distribution of tomosyn *in vivo*. However, the immunoreactivity of SNAP25 was in the axon and somata, suggesting the polarity of ES-AVP neurons *in vitro* were not completely established.

We used the ROCK inhibitor Y27632 in SFEBq/gfCDM cultured aggregates prior to dispersion. It has been reported that the selective ROCK inhibitor Y27632 can increase survival and cloning efficiency of dissociated single human embryonic stem cells [37], and that Y27632 decreases the amount of dissociation-induced apoptosis in mouse ES cells [38]. Sakisaka et al. reported that ROCK phosphorylates syntaxin-1 and forms a stable tomosyn-1 complex [39]. Therefore, Y27632 could block the binding of tomosyn-1 with syntaxin/SNAP25. However, Sakisaka et al. further assessed the phosphorylation of syntaxin-1 after the incubation with Y27632 for 30 minutes. Neurite outgrowth assays were performed after the incubation with Y27632 for 24 hours [39]. In the present study, we applied Y27632 only once when SFEBq/gfCDM cultured aggregates were dispersed on day 31, and we analyzed ES-AVP on days 39–40. Therefore the time course after Y27632 treatment in our study was much longer than that of Sakisaka et al., and the application of Y27632 showed little influence on our results, including on AVP release.

Overexpression of tomosyn-1 significantly reduced KCl-stimulated AVP secretion. In contrast, downregulation of tomosyn-1 with siRNA significantly increased KCl-stimulated AVP secretion. However, tomosyn-1 did not affect AVP secretion in basal conditions without KCl stimulation. These results demonstrated that tomosyn-1 negatively regulates the depolarization-evoked AVP secretion in mES-AVP cells. The negatively regulated effect of tomosyn-1 was consistent with previous reports that tomosyn-1 inhibits Ca^2+^-dependent release [26, 40]. It has been reported that insulin as well as neurotransmitters are negatively regulated by tomosyn-1 [32, 34–35, 40–41]. Therefore, the negative role of tomosyn-1 in AVP secretion was similar to that reported for neurotransmitter and insulin secretion. However, whether the dual role of the domain of the N-terminal WD40 repeats and C-terminal VLD in tomosyn-1 in neurotransmitter release is also involved in the molecular mechanism of AVP secretion needs to be clarified. In addition, although our results with overexpression or the knockdown (KD) experiments suggested tomosyn-1 was required and was sufficient for regulation of AVP secretion, because tomosyn-1 overexpression or KD had limited effects on AVP secretion, it was thought that tomosyn-1 is not a major regulator of the exocytosis process. However, in order to evaluate the contributions of tomosyn-1 in AVP release, we need to improve the ES-AVP cell culture systems in terms of the capacity of AVP release. *In vivo*, plasma AVP concentration increased more than several times in response to stimuli, including dehydration. However, the depolarization-evoked AVP secretion in ES-AVP cells in the present study was approximately two times as compared with controls.

Further studies are needed to clarify the significance of tomosyn-1 *in vivo*, for example, using knockout mice. In humans, CDI is characterized by decreased release of AVP, resulting in polyuria. Importantly, the etiological diagnosis remains unknown in about half of the patients with CDI [42]. An important next step would be to screen for gene mutations in tomosyn-1 in patients with idiopathic CDI. We anticipate our methods of mES-AVP culturing to provide a more sophisticated *in vitro* model of secretion of AVP, including application to studies of CDI-specific human samples and induced pluripotent stem cells.

## Materials and Methods

### Animals

Male Sprague-Dawley rats (7–9-weeks-old; body weight, 250–300 g; Chubu Science Materials, Nagoya, Japan) were housed two per plastic cage under controlled conditions (23.0 ± 0.5°C; lights on from 0900 to 2100) and provided with standard rat chow *ad libitum*. All procedures were performed in accordance with the institutional guidelines for animal care at Nagoya University, which conform to the National Institutes of Health animal care guidelines. All experimental protocols were approved by the Nagoya University Experiment Committee.

### Plasmid constructions

SNAP25 (clone ID; 4504644) from the RIKEN mouse FANTOM clone was obtained through DANAFORM (Yokohama, Japan). Tomosyn-1 cDNA (clone ID; MC223621) and PCMV-Entry Vector (clone ID; PS100001) were purchased from Origene (Rockville, MD, USA). Tomosyn-1-specific siRNA and scrambled siRNA were procured from Sigma-Aldrich (St. Louis, MO, USA). The following siRNA sequences were used: scrambled, #1 (5'-CAGUCGCGUUUGCGACUGG-3'); Tomosyn-1 #1 (5'-GUACUAUAUUGAGGUUAAATT-3'); and tomosyn-1 #2 (5'-CAGUUGUGCAUAUAAGUGATT-3').

In this manuscript, tomosyn-1 refers to syntaxin binding protein 5, and tomosyn-2 refers to syntaxin binding protein 5-like, whereas tomosyn refers to both paralogs.

### Antibodies

The primary antibodies used in this study are listed in Table 1. Anti-tomosyn antibodies (sc-13610 and ab41271) are pan-specific (tomosy-1/2) that recognize both isoforms.

**Table 1:** List of primary antibodies.

Protein	Catalog No	Poly/Mono	Host	IB	IHC	ICC	IP	Company
Tomosyn	sc-136105	Mono	Mouse	1:200		1:50	5μg	Santa Cruz Biotechnology
	ab41271	Poly	Rabbit	1:1000	1:1000	1:100		Abcam
SNAP25	ab5666	Poly	Rabbit	1:1000		1:50		Abcam
	ab41455	Poly	Rabbit		1:20			Abcam
	111011	Mono	Mouse	1:1000			5μg	Synaptic Systems
Syntaxin1A	110111	Mono	Mouse	1:2000			5μg	Synaptic Systems
	110302	Poly	Rabbit		1:200			Synaptic Systems
Syntaxin1B	110403	Poly	Rabbit	1:1000	1:1000		5μg	Synaptic Systems
Copeptin	sc-7812	Poly	Goat		1:100	1:100		Santa Cruz Biotechnology
AVP	AB1565	Poly	Rabbit	1:1000				CHEMICON
β-actin	A2228	Mono	Mouse	1:10000				Sigma
Akt	4691	Mono	Rabbit	1:10000				Cell Signaling
N-cadherin	610920	Mono	Mouse	1:2000				BD Bioscience
normal mouse IgG	sc-2025	Mono	Mouse				5μg	Santa Cruz Biotechnology
normal rabbit IgG	PP64	Poly	Rabbit				5μg	Millipore
NeuN	MAB377	Mono	Mouse			1:50		Millipore

Poly/Mono = polyclonal or monoclonal antibody; IB = immunoblot dilutions used; IHC = immunohistochemistry dilutions used; ICC = immunocytochemistry dilutions used; IP = immunoprecipitation total protein used.

### Recombinant proteins

Plasmid for GST-SNAP25 was constructed using the Gateway system (Invitrogen, Carlsbad, CA, USA). In brief, we first sub-cloned the cDNA fragment into the pENTR-D-TOPO vector, followed by recombination into a destination vector (pDEST^TM^15; Invitrogen) with LR clonase II (Invitrogen). GST-SNAP25 was expressed in *Escherichia coli* (Rossetta DE3) cells and purified by glutathione Sepharose 4B chromatography (GE Healthcare UK Ltd, Hertfordshire, UK). GST-cdc42-GTPγS was provided by Dr. K. Kaibuchi.

### Rat posterior pituitary lysates

Fresh rat posterior pituitary were isolated and solubilized in 500 μL of lysis buffer [10 mM HEPES-NaOH (pH 7.4), 0.5% NP40, 1 mM CaCl_2_, 50 mM NaCl, and 5 mM MgCl_2_]. The suspension was homogenized and sonicated. The homogenate was centrifuged at 43,000 × rpm for 30 min at 4°C and the supernatant was collected.

### *In vitro* binding assays

Rat posterior pituitary lysates were precleared with glutathione Sepharose 4B for 1 h at 4°C, and thereafter the lysates were mixed with GST-SNAP25 or GST-cdc42-GTPγS, and glutathione Sepharose 4B overnight at 4°C. The sediment was separated and washed four times with wash buffer [10 mM HEPES-NaOH (pH 7.4), 1 mM CaCl_2_, 50 mM NaCl, and 5 mM MgCl_2_] and bound proteins were eluted in SDS sample buffer.

### In-gel digestion and MS analyses

Eluted samples were loaded onto gels for immunoblotting, separated by SDS-PAGE, and subjected to silver staining using 2D-Silver Stain-II (Cosmo Bio Co., Ltd., Tokyo, Japan). The gel bands were then excised and sent to MBL (MBL, Medical & Biological Laboratories Co., Nagoya, Japan) for in-gel digestion and nano LC-TOF MS/MS analysis. Data were analysed using ProteinPilot™ (AB SCIEX, Framingham, MA, USA) to search the Swiss-Protein database.

### Immunohistochemistry

Rats were sacrificed under deep ether anesthesia by transcardiac perfusion with 4% paraformaldehyde in phosphate-buffered saline (PBS). The brains were removed, postfixed in 4% paraformaldehyde in PBS, and cryoprotected, and cryostat sections (7 μm) were made as previously described [43]. The sections were incubated in 0.3% Triton-X100 for 15 min, treated with 50 mM glycine in PBS for 15 min for quenching, blocked with 5% bovine serum albumin (BSA) for 1 h, and incubated with primary antibodies overnight. After three washes with PBS, the sections were incubated for 1 h at room temperature with 4’,6-diamidino-2-phenylindole (DAPI) (Dojindo Laboratories, Kumamoto, Japan) and the following secondary antibodies: Alexa Fluor^®^ 488- and 594-conjugated anti-IgG antibodies (Invitrogen; 1:1000). After an additional three washes with PBS, cells were mounted with anti-fade solution (ProLong^®^ Gold, Life Technologies, Foster City, CA, USA). All fluorescently stained sections were examined with a fluorescence microscope (BZ X710 and BZ-8000; Keyence, Osaka, Japan).

### Western blotting analyses

Lysates were subjected to one-dimensional SDS-PAGE. Gels were transferred to nitrocellulose membranes and then blocked with 4% skim milk. The primary antibodies were incubated with the membranes overnight. The membranes were washed with PBS/Tween and then incubated with horseradish peroxidase (HRP)-conjugated antibodies against mouse or rabbit IgG (1:1000) for 1 h. After three washes with PBS/Tween, the membranes were incubated with ECL^TM^ Western Blotting Detection reagent (GE Healthcare) and exposed to X-ray film (Cosmo Bio Co., Ltd.).

### Subcellular fractionation

Subcellular fractionation was performed using the ProteoExtract^®^ Subcellular Proteome Extraction Kit (Calbiochem, San Diego, CA, USA) according to the manufacturer’s instructions. In brief, cytosolic proteins from rat posterior pituitary were released with Extraction Buffer 1 (Calbiochem). Subsequently, membranes and membrane organelles were solubilized with Extraction Buffer 2 (Calbiochem).

### Quantitative RT-PCR

After decapitation, the hypothalamus and cortex were removed and stored at -80°C until use. SFEBq/gfCDM cultured ES cell aggregates were washed with PBS once and collected. Total RNA was extracted using an RNeasy Mini Kit (QIAGEN, Valencia, CA, USA), following the manufacturer’s instructions. One μg of RNA was reverse-transcribed using PrimeScript RT Master Mix (Takara Bio Inc., Otsu, Japan) in a final volume of 25 μL. Real-time PCR was performed on the reverse transcription products using SYBR Premix Ex Taq II (Takara Bio, Inc.) and an Applied Biosystems StepOne™ Real-Time PCR System (Applied Biosystems), with cycling parameters of 40 cycles of 95°C for 15 s and 60°C for 60 s, following the manufacturer’s instructions. The primers used for this experiment were specifically designed and synthesized by Life Technologies. As previously described [40], the following primers were used: rat m-tomosyn-1 forward (5′-CTCCGACTTCCGCAAAGATGTC-3′), rat m-tomosyn-1 reverse (5′-TTCAGCGTGATGACAAAGGC-3′); mouse m-tomosyn-1 forward (5′-CTCCCACTTCCGCAAAGATGTC-3′), and mouse m-tomosyn-1 reverse (5′- TTCAGCGTGATGACAAACGC-3′). All samples were run in duplicate. The mRNA levels were compared using the Ct-method, which was normalized to β-actin. In each experiment, a negative (RT minus) control was included.

### Coimmunoprecipitation assays

Rat posterior pituitary lysates were precleared with protein A/G Sepharose (GE Healthcare) or pre-clearing matrix (Santa Cruz Biotechnology, Santa Cruz, CA, USA) for 1 h at 4°C. Pre-cleared lysates were incubated with 10 μg of control IgG or IP antibodies at 4°C followed by additional incubation with protein A/G Sepharose for 3 h at 4°C. Protein A Sepharose was used for mouse IP antibodies and protein G Sepharose was use for rabbit IP antibodies. The IP matrix (Santa Cruz Biotechnology) was incubated with 5 μg of control IgG (1 μg) or IP antibodies overnight at 4°C followed by additional incubation with pre-cleared lysates overnight at 4°C. Thereafter, the sediments were washed four times with the wash buffer used in the *in vitro* binding assays, and bound proteins were eluted in SDS sample buffer.

### Differentiation of mouse ES cells into AVP secreting neurons

Mouse ES cells differentiated into rostral hypothalamic progenitor cells, including AVP secreting neurons. Dissociated mES cells quickly reaggregated and were cultured as floating aggregates, and as serum-free cultures of embryoid body-like aggregates (SFEBq), in growth factor-free chemically defined medium (gfCDM) [25]. Using this method, mouse ES cells (EB5) were maintained in medium containing DMEM/F12 supplemented with 1% fetal calf serum (FCS), 10% knockout serum replacement (KSR), 2 mM glutamine, 0.1 mM nonessential amino acids, 1 mM pyruvate, 0.1 mM 2-mercaptoethanol, and 2000 U/mL leukemia inhibitory factor (LIF). For SFEBq differentiation, ES cells were dissociated to single cells using TrypLE™ Express enzyme treatments (Life Technologies) and rapidly aggregated in gfCDM using 96-well, low cell adhesion plates (Lipidure^®^-coated wells; NOF, White Plains, NY, USA). The gfCDM contained Iscove’s modified Dulbecco’s medium/Ham’s F-12 in a 1:1 ratio, chemically defined lipid concentrate, penicillin/streptomycin, monothioglycerol (450 μM), purified BSA, and human apotransferrin (15 μg/mL). Each well contained 3,000 cells per 100 μL gfCDM. The day on which ES cells were transferred to Lipidure^®^-coated wells for reaggregation was defined as differentiation day 0. On day 10, 100 μL of DFNB medium was added to each well. DFNB medium contains DMEM/F12 supplemented with 7 g/L glucose, 1× N2 supplement (50× stock, Gibco-BRL, Grand Island, NY, USA) and 1× B27 (50× stock, Gibco-BRL), and 10 ng/mL ciliary neurotrophic factor (CNTF; R&D systems, Minneapolis, USA) for further neuronal differentiation. Aggregated cells were transferred into 6-well Transwell^®^ culture inserts (Corning, Corning, NY, USA) at eight aggregates per well on day 13. Aggregates were maintained in DFNB medium, and one-half volume of DFNB medium was changed every other day for up to day 31 of culture. SFEBq/gfCDM-cultured ES cell aggregates at day 31 were dispersed into 60-mm dishes using the Nerve-Cell Dissociation Media Kit (MBX9901; DS Pharma Biomedical Co, Osaka, Japan) according to the manufacturer’s instructions. Dispersed aggregates were plated at a density of 1.0 × 10^6^ cells per 60-mm tissue culture dish. Prior to dispersion, a rho-associated, coiled-coil containing protein kinase (ROCK) inhibitor (Y27632; Wako, Osaka, Japan) was added to SFEBq/gfCDM cultured aggregates at a concentration of 10 μM and incubated at 37°C for 15 min.

### Immunofluorescence microscopy

Dissociated SFEBq/gfCDM cultured cells were washed with PBS once and fixed with 4% paraformaldehyde for 15 min. Subsequent procedures were performed, including immunohistochemistry, and samples were analysed using confocal laser scanning microscopy (A1Rsi; Nikon, Tokyo, Japan).

### siRNA transfection

Dissociated SFEBq/gfCDM cultured cells were transfected with mouse tomosyn-1 siRNA or scrambled siRNA using Lipofectamine^®^ RNAiMax Transfection reagent (Life Technologies). At 48 h after transfection, cell lysates were subjected to immunoblotting to evaluate the effects of siRNA.

### Overexpression of tomosyn-1

Dissociated SFEBq/gfCDM cultured cells were transfected with mouse m-tomosyn-1 vector or empty vector using Lipofectamine^®^ 3000 Reagent (Life Technologies). At 48 h after transfection, cell lysates were subjected to immunoblotting to evaluate the effects of overexpression as well as siRNA transfection.

### Analysis of AVP release

Dissociated SFEBq/gfCDM cultured cells were washed twice with PBS and preincubated in artificial cerebrospinal fluid (aCSF) consisting of 124 mM NaCl, 3 mM KCl, 26 mM NaHCO_3_, 2 mM CaCl_2_, 1 mM MgSO_4_, 1.25 mM KH_2_PO_4_, and 10 mM D-glucose at pH 7.4 for 10 min at 37°C. Cultured cells were then incubated in either high potassium (100 mM) aCSF consisting of 28 mM NaCl, 99 mM KCl, 26 mM NaHCO_3_, 2 mM CaCl_2_, 1 mM MgSO_4_, 1.25 mM KH_2_PO_4_, and 10 mM D-glucose at pH 7.4, or control aCSF for an additional 30 min. Each incubated solution was individually frozen, and AVP concentration in the medium was measured with a radioimmunoassay (RIA) kit (AVP-RIA kit; Mitsubishi Chemical, Tokyo, Japan), as described previously [44].

### Statistical Analyses

Results are expressed as the mean ± SEM, and statistical analyses were performed using Student’s *t*-tests or one-way ANOVA followed by Fisher’s projected least significant difference test as indicated in the figure legends. P values less than 0.05 were considered to be significant.

## Supporting Information

S1 Fig SNAP25, glutathione S-transferase (GST) pulldown assays using GST-SNAP25 and rat posterior pituitary lysates. The eluted proteins were subjected to sodium dodecyl sulphate polyacrylamide gel electrophoresis (SDS-PAGE) followed by silver staining. A band migrating at a molecular weight of approximately 130 kDa was detected in the eluted sample (right panel, boxed and marked with the arrow). The band migrating at a molecular weight of approximately 130 kDa was not detected in the eluted samples using GST-cdc42-GTPγS beads with rat posterior pituitary lysates (left panel), strongly suggesting that nonspecific binding of proteins in the lysates to the beads or GST tag did not occur. Marker = molecular weight markers.
(EPS)

S2 Fig Tomosyn or SNAP25 was not detected in the SON or PVN. The cryosections of SON and PVN were double stained with anti-tomosyn, anti-SNAP25, or anti-copeptin antibody. Copeptin is stained as red; tomosyn and SNAP25 are green. Single immunostaining for tomosyn, SNAP25, and copeptin in SON and PVN are shown. SON = supraoptic nucleus; PVN = paraventricular nucleus.White scale bars indicate 100 μm (white bars) or 200 μm (white dotted bars).
(EPS)

S3 Fig Tomosyn-1 did not significantly affect AVP secretion in the absence of KCl stimulation. AVP secretion of artificial cerebrospinal fluid cultured cells (aCSF) (non-treated, NT; n = 8), empty vector (Vector; n = 6), tomosyn-1 vector (Tomosyn-1; n = 7), scrambled siRNA (siScr; n = 8), siTomosyn-1 #1 (n = 8), and siTomosyn-1 #2 (n = 8) in the absence of KCl stimulation, are indicated as the ratio to the control (NT). Values are expressed as the mean ± SEM.
(EPS)
